# Deep Learning Approach for Automatic Microaneurysms Detection

**DOI:** 10.3390/s22020542

**Published:** 2022-01-11

**Authors:** Muhammad Mateen, Tauqeer Safdar Malik, Shaukat Hayat, Musab Hameed, Song Sun, Junhao Wen

**Affiliations:** 1 Department of Computer Science, Air University Multan Campus, Multan 60000, Pakistan; muhammad.mateen@aumc.edu.pk (M.M.); tauqeer.safdar@aumc.edu.pk (T.S.M.); 2 Department of Computer Science, Iqra National University, Peshawar 25000, Pakistan; s.hayat@inu.edu.pk; 3 Department of Electrical & Computer Engineering, Sahiwal Campus, COMSATS University Islamabad, Sahiwal 57000, Pakistan; musabhameed@cuisahiwal.edu.pk; 4 School of Big Data & Software Engineering, Chongqing University, Chongqing 401331, China; sun2007song@cqu.edu.cn

**Keywords:** convolutional neural networks, diabetic retinopathy, feature embedding, microaneurysms detection

## Abstract

In diabetic retinopathy (DR), the early signs that may lead the eyesight towards complete vision loss are considered as microaneurysms (MAs). The shape of these MAs is almost circular, and they have a darkish color and are tiny in size, which means they may be missed by manual analysis of ophthalmologists. In this case, accurate early detection of microaneurysms is helpful to cure DR before non-reversible blindness. In the proposed method, early detection of MAs is performed using a hybrid feature embedding approach of pre-trained CNN models, named as VGG-19 and Inception-v3. The performance of the proposed approach was evaluated using publicly available datasets, namely “E-Ophtha” and “DIARETDB1”, and achieved 96% and 94% classification accuracy, respectively. Furthermore, the developed approach outperformed the state-of-the-art approaches in terms of sensitivity and specificity for microaneurysms detection.

## 1. Introduction

**Figure 1:**
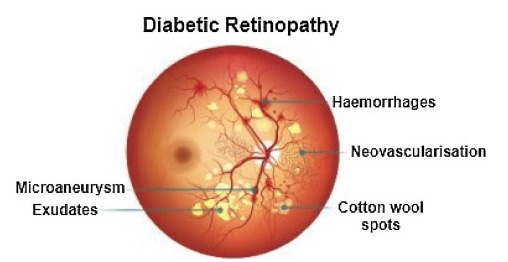
Diabetic retinopathy with microaneurysms.

Recently, in the area of ophthalmology, diabetic retinopathy (DR) has been considered as a most common and serious disease, affecting the eyesight and leading towards blindness or complete vision loss. According to the survey report, many experts have declared that almost 90% of diabetic patients can be saved through early identification of DR [1]. Diabetic retinopathy encompasses retinal abnormalities including cotton wool spots, hemorrhages, exudates, and microaneurysms (MAs), which lead to non-reversible blindness and vision impairment. In the case of microaneurysms, MA is an early sign of DR, and is considered as a basic element of DR. MAs are very small in size and mostly skipped by the ophthalmologists because of manual detection techniques. In the case of an automatic approach, early and accurate detection of MAs is possible, which can help the ophthalmologists to control diabetic retinopathy at a very early stage. As a result, the affected patient can start treatment in time and can save oneself from the worst level of DR, which leads to complete vision loss [2]. Figure 1 shows diabetic retinopathy with the signs of MAs.

In diabetic retinopathy, microaneurysms detection and classification is a difficult task due to different features, including texture, color, and size on fundus images. Hence, in the field of ophthalmology, the advancement of reliable and fast automatic diabetic retinopathy detection tools is the most considerable area of research. In recent research work, few studies were encountered related to reliable microaneurysms detection and classification. Microaneurysms evaluation techniques are divided into supervised, unsupervised, and mathematical morphologically based operations. 

Habib et al. [3] introduced an automated microaneurysms detection and classification application based on retinal fundus images. The feature extraction was based on the Gaussian matched filter and then extracted features were fed towards the ensemble classifier to classify the microaneurysms points on based on pixels. The receiver operating curve (ROC) of the reported approach was 0.415. The deficiency of the reported approach was that it ignored the issue of over fitting, as well as could not follow the principle of standard feature selection. Similarly, feature extraction for microaneurysms was also applied using the Gaussian filter approach [4]. Further processing was incorporated using the green channel to identify the location of the microaneurysms region. In the end, the segmentation of microaneurysms achieved 93.41% of accuracy on the basis of the Gaussian matched filter. Furthermore, maximum and canny edge entropy-based approaches were applied to identify the microaneurysms using retinal fundus images [5]. Image normalization technique was applied to remove the vessel area, and after that, segmentation of microaneurysms was based on entropy and canny edge approaches. Moreover, erosion-based morphological operation was applied to identify the patterns of microaneurysms with 90% accuracy. Microaneurysms detection was also performed using Gaussian, kirsch, and median filters to extract the microaneurysms features on the basis of the green channel of colored image [6]. The extracted features were fed into a multi-agent framework for discrimination of true microaneurysms pixels from the false pixels.

Recently [7], several image processing approaches, including feature extraction and contrast enhancement, were applied to introduce novel concepts for the development of microaneurysms detection and classification. Wu et al. [8] introduced profile and local features to identify the microaneurysms. Initially, pre-processing was applied to fundus photographs to overcome the image artifacts, including noise, to improve the microaneurysms detection performance. Moreover, for the classification of microaneurysms pixels, a k-mean-based classifier was used and achieved a ROC of 0.202. However, the reported approach could not obtain a better performance in the case of too noisy fundus images.

Romero et al. [9] introduced hit-or-miss and bottom-hat transform techniques for microaneurysms detection after a pre-processing technique was applied to the gray content of images. In the process of getting an intact reddish region, a bottom-hat transformation approach was implemented on the gray content of fundus images. Furthermore, the hit-transformation technique was applied to eliminate the blood vessels. Finally, radon transform and principal component analysis were used to identify true microaneurysms points with 95.93% classification accuracy. Moreover, an average-based filter and top-hat transformation were implemented and obtained 100 specificities for the removal of retinal artifacts [10]. Then, a multi-overlapping window and radon transform were applied for the cleavage of microaneurysms, which obtained 94% sensitivity. Hence, there were few drawbacks of the reported approach, through which microaneurysms points were difficult to locate in the case of low-contrast microaneurysms pixels and the approach was also computationally expensive. To overcome these issues, location-based contrast enhancement and naïve Bayes classifiers [11,12] were applied and obtained better microaneurysms results.

A coarse segmentation technique [11] was considered to firstly identify the microaneurysms candidate region. Furthermore, extracted microaneurysms features were classified and discriminated true microaneurysms points from false microaneurysms points using naïve Bayes with 82.64% sensitivity and 99.99% accuracy. However, a few deficiencies were noted, including the fact that it was computationally expensive as well as limited for blur and faint microaneurysms pixels. CLAHE technique was applied to overcome the issues highlighted to identify blur and faint microaneurysms pixels [11]. In the reported approach, the fundus images were divided into contextual pieces to identify the low-contrast and tiny microaneurysms pixels with 85.68% sensitivity. The reported technique was used to evaluate 47 fundus images to generalize the other related medical diseases. A sparse representation classifier [13] was implemented on the basis of dictionary learning to identify the microaneurysms pixels. Firstly, microaneurysms region localization was incorporated by the Gaussian correlation filter. Furthermore, microaneurysms points and non-microaneurysms points were classified using a sparse representation classifier with microaneurysms features as inputs, which obtained an improved rate of sensitivity of 0.8%.

Akram et al. [14] introduced an automatic microaneurysms detection approach to grade the fundus images, regardless of whether images have microaneurysms regions or not. The performance accuracy of the reported approach was noted as 99.40%. The implemented approach was considered as computationally expensive; however, classification performance for the discrimination of the extracted features was admirable. In this case, ensemble-based adaptive and sparse principle component analysis techniques decreased the number of false-positive values in the microaneurysms detection and had also removed the class imbalance issue commonly accrued in the fundus images with the performance of AUC as 0.90 [15,16]. Javidi et al. [17] applied two dictionaries and morphological operations for microaneurysms identification on the basis of retinal fundus images. Firstly, a Morlet-based wavelet technique was applied to localize the microaneurysms candidate region. Then, the evaluation was performed to classify the microaneurysms regions using extracted features on the basis of binary dictionaries. The reported approach achieved an ROC of 0.267. Furthermore, Yadav, D. et al. [18] presented a novel approach with low computational power to detect the microaneurysms using a machine learning classifier. The performance of the developed approach was measured with various classification techniques and obtained highest accuracy (83.33%) using random forest.

However, the approach was considered computationally expensive in addition to its failure to identify the microaneurysms pixel in the case of poor contrast and faint retinal fundus images. Shan et al. [19] applied a deep learning-based approach, namely stacked sparse auto-encoder (SSAE), to detect the microaneurysms. In the very early stage of the reported approach, images were divided into small pieces called patches. In the next step, the feature extraction was applied using the SSAE approach to learning about the specified features on the basis of patches. In the end, patches were classified according to the specified features to distinguish between true microaneurysms and false microaneurysms using softmax classifiers with 96.2% AUC. Furthermore, Derwin, D. J. et al. [20] introduced a texture-based microaneurysms classification approach and obtained a 0.421 score of receiver response using ROC database. Additionally, Derwin, D. J. et al. [21] developed an advanced feature extraction approach for the classification of microaneurysms using a local neighborhood differential coherence pattern based on a feed-forward neural network. The performance of the novel approach was measured based on free-response receiver operating characteristics, obtaining a 0.481 score for ROC.

In diabetic retinopathy detection, microaneurysms and hemorrhage segmentation has been performed using a two-stage approach, namely multiple kernel filtering [22]. In the reported approach, a patch-based analysis technique was adopted instead of full retinal images for the analysis of retinal lesions of various sizes. A support vector machine was implemented to classify the retinal microaneurysms and hemorrhage candidates from the other regions of fundus images with an AUC of 0.97. Moreover, Du, J. et al. [23] developed a microaneurysms detection approach based on feature extraction and classification. In the reported approach, block filtering and local minimum region extraction is performed during the candidate extraction stage and the microaneurysms classifier is trained in the second stage using a series of descriptors. The performance of the proposed approach is measured on the basis of FROC metric, and a score of 0.516 was obtained the E-Ophtha database.

Furthermore, another technique using machine learning was presented by Adal et al. [24] to identify the microaneurysms. Initially, fundus images were normalized with pre-processing techniques, and after that, feature extraction was performed by various scale descriptors. In the end, the semi-supervised technique was implemented for the training of extracted features to distinguish microaneurysms pixels from non-microaneurysms pixels with an AUC of 0.36. Moreover, various approaches failed to overcome the challenges related to over fitting for large-scale datasets, as well as having a low scale of discriminative power [19,22,24]. In the identification of microaneurysms, DR, Y.-H. Li, et al. [25] designed an automatic diagnosis application on the basis of machine learning. Additionally, the authors introduced a detection and classification system for diabetic retinopathy on the basis of convolutional neural networks. Furthermore, S. Suriyal et al. [26] designed a mobile-based real-time assistance application of diabetic retinopathy through a deep learning approach. In this application, the tensor flow deep neural network model was applied to train and validate the fundus images. Furthermore, Jadhav, M. et al. [27] presented a Gabor filter-based approach for early identification of microaneurysms and achieved results with an Indian dataset in terms of sensitivity, specificity, and accuracy as 76.75%, 78.76%, and 80.85% respectively.

Moreover, the R-CNN-based microaneurysms detection approach was introduced in another study. Using the reported approach, features were extracted using VGG16 and classification was performed to classify the candidate regions [28]. Using another approach, microaneurysms were detected using statistical and Stockwell transform features to distinguish the normal and affected retinal images. In total, 1140 retinal images were used in the training and testing phases [29]. Furthermore, mathematical morphological operations were applied to detect the microaneurysms using fundus images. In the reported study, three models were introduced, namely an image enhancement approach, removal of artifacts including blood vessels, and, finally, extracted features were analyzed for the selection of microaneurysms candidate among other features. In addition, Joshi, S. and Karule, P. [30] presented a novel approach based on three major components, including image enhancement, extraction of blood vessels, and MA candidate selection. The reported approach achieved 92% accuracy using the DIARETDB1 dataset. Moreover, Melo, T. et al. [31] developed a sliding band filter for microaneurysms detection using retinal fundus images. The combination of shape, contrast, and color of retinal fundus images were classified by ensemble classifiers. The accuracy of the reported approach was analyzed using four datasets, including E-Ophtha microaneurysms. Furthermore, Liao, Y. et al. [32] presented a novel approach for the detection of microaneurysms using fundus images. The presented approach is based on the use of encoder–decoder algorithms to locate the signs of microaneurysms for the accurate and early detection of microaneurysms. The performance of the developed approach was analyzed using E-Ophtha-MA and Retinopathy Online Challenge datasets.

The traditional machine learning approaches namely support vector methodologies, for the classification of medical images began long ago. However, these techniques have the following limitations: In recent years, the performance has been far from the practical standard, and the development of them has slowly ceased. Furthermore, feature selection and extraction are time-consuming processes and vary according to various objects. Alternatively, deep learning methods, especially the convolutional neural networks (CNNs), are widely used in image classification tasks and have obtained significant results since 2012 [33]. In the proposed methodology, we have adopted the Inception-v3 model as the feature extractor, because of its outstanding performance and good generalization ability, and also used VGG19 architecture, as VGG19 is an advanced CNN with pre-trained layers and a great understanding of what defines an image in terms of shape, color, and structure. This architecture, with small filter sizes, is very deep and has been trained on millions of diverse images with complex classification tasks. Furthermore, to optimize the extracted features, the feature embedding approach has been applied, because feature embedding is an emerging research area that intends to transform features from the original space into a new space to support effective learning. Moreover, embedded learning is a domain-sensitive technique with the output depending on the underlying data; embedded features are context sensitive and can often capture the data correlations in the original feature space.

The pipeline of the proposed framework is presented as follows: Firstly, the data pre-processing is performed to discriminate the microaneurysms using a grayscale image conversion and shade correction approach. Secondly, the data patch generation is performed. In the next phase, patch-based images are fed into VGG-19 and Inception-v3 for feature extraction. After completion of feature extraction from both pre-trained models, the extracted features are embedded through the feature embedding technique. In this phase, the cross-entropy loss is calculated and weighted through the back-propagation approach. Finally, the feature vector is fed into the softmax classifier for the final decision. The performance of the proposed methodology was evaluated through publicly available datasets, namely “E-Ophtha” and “DIARETDB1”, and obtained better classification results than the existing approaches to classify the microaneurysms for DR.

The major contribution of the manuscript is to propose a novel and efficient deep-CNN-based approach to solve the problem of microaneurysms detection and classification, utilizing the strength of deep selective fused features using the feature embedding approach. The proposed framework makes our approach different from the previous methodologies for microaneurysms detection and classification. Furthermore, this framework provides an edge in terms of sensitivity, specificity, and accuracy compared to the state of the art approaches.

The remaining sections of the article are managed as follows: Section 2 presents the research methodology, Section 3 explains the experimental setup, then experimental results are discussed in Section 4, and finally, Section 4 covers the conclusions.

## 2. Materials and Methods

**Figure 2:**
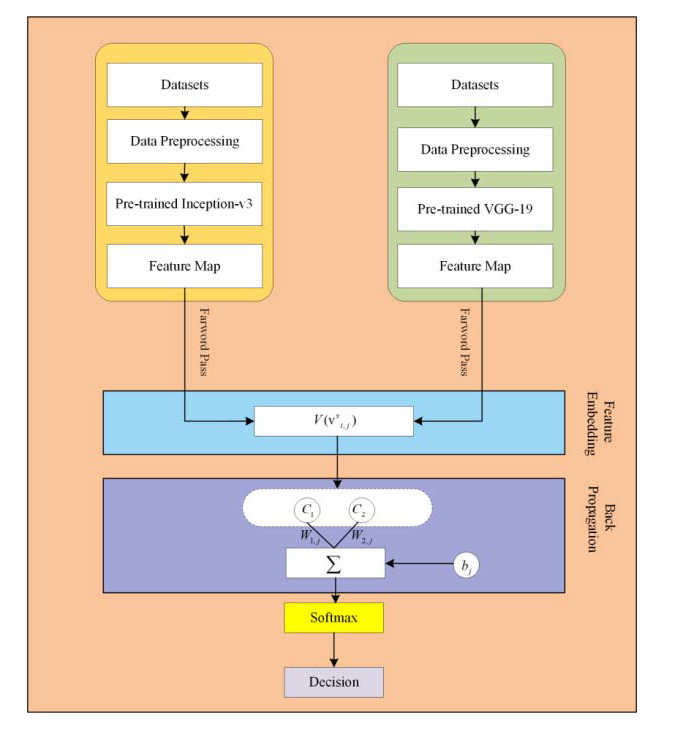
The block diagram of the proposed approach.

In the proposed framework, pre-trained CNN based microaneurysms detection for diabetic retinopathy has been performed using a feature embedding approach. Figure 2 demonstrates the block diagram of the proposed approach.

### 2.1. Datasets

Data selection is an essential part of choosing the data according to the proposed technique for experiments. E-Ophtha and DIARETDB1 are two publicly available databases that were applied to experiments of the proposed technique to identify the microaneurysms. E-Ophtha dataset has two subsets, one is about exudates and the other one is related to microaneurysms. “E-Ophtha MA” contains 148 microaneurysms photographs, observed by four experts of ophthalmology [34]. The quality of retinal photographs varies from 1400 × 960 to 2544 × 1696 pixels. Similarly, DIARETDB1 contains 89 fundus photographs with 1500 × 1152 pixels of resolution [35].

**Figure 3:**
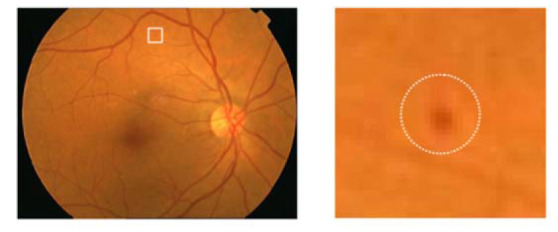
Encircled mark of the microaneurysms taken from the original fundus image.

A specified digital camera with a 50-degree field of view was used to take all the fundus images of both retinal datasets. The experts of ophthalmology examined the microaneurysms in diabetic retinopathy. All the retinal images were organized to resize every fundus image according to the standard size of DIARETDB1 images, specifically 1500 × 1152 pixels of resolution. The estimated scale size of the image was determined on the basis of the standard size of the retinal optic disc. The sample taken from affected retinal images is shown in Figure 3.

### 2.2. Data Pre-Processing

**Figure 4:**
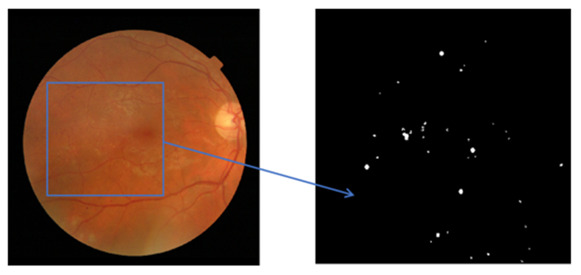
The input retinal fundus image and the background retinal fundus image.

Diabetic retinopathy is a retinal disease with various features, including hemorrhages, exudates, and microaneurysms. In the case of specified retinal feature detection, the dataset requires data pre-processing to make a standard feature selection of diabetic retinopathy to discriminate it from noisy data. Data pre-processing is a procedure to apply the optimum strategy to distinguish the optimal DR or non-DR. Hence, data pre-processing is performed on the raw fundus images before feature extraction. In diabetic retinopathy, exudates are considered as bright lesions, but dark lesions are referred to as microaneurysms. In this scenario, it is important to apply data pre-processing to identify the dark lesions for microaneurysms. In the data pre-processing, different kinds of algorithmic approaches are performed, namely the grayscale image conversion approach to achieve better contrast value. Meanwhile, a shade correction approach is applied to approximate the photographs and further subtract from the original image. Figure 4 shows the information extracted from retinal fundus images in the background form.

In the right side background image, an optimal form of discrimination can be found. In this approach, the adaptive learning rate (ALR) obtained excellent progress to discriminate against the retinal features in the region of interest (ROI). Furthermore, a significant approach in the context of microaneurysms discrimination for diabetic retinopathy has been introduced by Antal, based on ensemble clustering [36]. 

### 2.3. Patch Generation 

**Figure 5:**
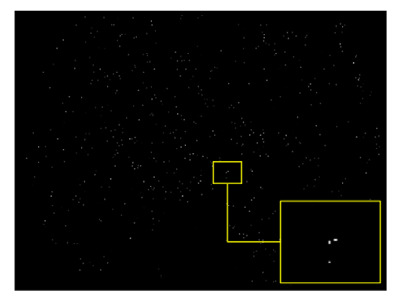
Example of 25 × 25 patch size of background images.

In this section, the data preparation was performed according to the proposed approach for experiments and was standardized due to the different sizes of microaneurysms. In the patch-based analysis, a 25 × 25 patch size of fundus images was used with two types of groups, MAs and non-MAs. In the retinal non-MA patch group, numerous retinal abnormalities were included, namely background tissues, retinal blood vessels, and optic nerve heads. In the patch generation phase, all the patches were obtained and extracted without any kind of an overlap, and Figure 5 represents the example of the MA patch.

### 2.4. Pre-Trained DNNs for Feature Extraction

In the proposed methodology, pre-trained deep neural network models are used for feature extraction. Furthermore, the adopted DNN architectures are joined with a fully connected layer for fundus image classification. In the case of combined features, there could be several kinds of features that define circularity, roundness, and compactness, achieved from single shape descriptor. In the context of image-based analysis, it was decided to select well-reputed deep neural network models, named visual geometry group network VGGNet-19 [37] and Inception-v3 [38], for feature extraction.

#### 2.4.1. VGG-19 DNN

**Figure 6:**
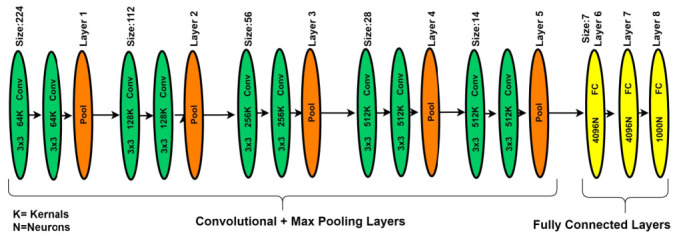
The VGG-19 model for patch-based analysis of microaneurysms.

In deep learning, different types of deep CNN models have been introduced. The visual geometry group network (VGGNet) model is one of the DNN models most commonly used for image analysis for feature extractions. The VGGNet model can be considered as the deep form of the AlexNet model because VGGNet is related to the AlexNet model, but with extra convolutional layers. The VGGNet model is expended on the behalf of kernel-sized filter replacement with a size of (3 × 3) filter windows, as well as (2 × 2) pooling layers, repeatedly. In the case of pre-trained operations, the VGG-19 model was implemented on the benchmark ImageNet dataset. Generally, the VGG-19 model has convolution layers, ratification layers, pooling, and FC layers, but through patch-based analysis, FC layers are removed and the extracted features are concatenated using a feature embedding technique with the feature extracted from the patch-based analysis [37]. In the literature study, the VGGNet-19 architecture better performed on deep features of image-based analysis as compared to AlexNet architecture. Figure 6 presents the basic architecture of the VGG-19 model.

#### 2.4.2. Inception-v3 DNN

**Figure 7:**
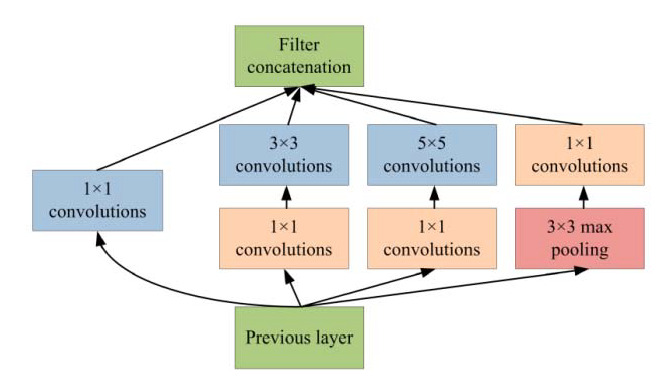
Inception-v3 model for patch-based analysis of microaneurysms.

Generally, Inception-v3 architecture is a deep neural network with convolutional, pooling, rectified, and fully connected layers. In the patch-based analysis of the proposed approach, fully connected layers are removed and the feature maps are concatenated using a feature embedding technique with the feature extracted from VGG-19 neural network. In the area of patch analysis, Inception-v3 architecture achieved outstanding performance in recognizing and classifying the input patch images. In the phase of patch-based analysis, the Inception-v3 model is used for feature extraction to spread out the numerous convolutional filters with different sizes to make an inventive single filter. Moreover, the inventive filter abates the number of parameters as well as decreases the overall computational complexity. Inception-v3 with heterogeneous-sized filters achieves better accuracy and low-dimensional embeddings. In the Inception-v3, the initial layer searches out the boundaries and, after that, the deepest layers help to recognize the microaneurysms for diabetic retinopathy. The composition of the Inception-v3 model is based on convolutional blocks, and the highest layer states the most complex functional mappings throughout the input and output variables after every convolutional layer, followed by the normalization [38]. Figure 7 shows the basic architecture of the Inception-v3 model.

### 2.5. Feature Embedding 

**Figure 8:**
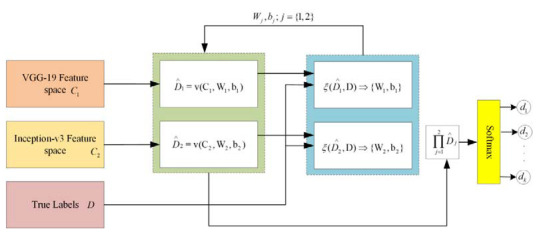
Implementation of feature embedding approach with gradient descent optimizer.

In the proposed framework, two different kinds of DNN are applied for feature extraction. Through patch-based analysis, pre-trained convolutional neural networks, including VGG-19 and Inception-v3 DNNs, are applied for feature extraction through fundus images. In the proposed framework, there is a significant task of applying the feature embedding technique after the feature extraction from both convolutional neural networks (CNNs). In this framework, the choice of feature embedding technique is used to merge the extracted features obtained from different CNN models for the improvement of classification accuracy. The basic purpose of feature embedding is to concatenate the extracted features obtained from different feature extractors and build a single feature vector, like $V=\{v_{1}^{y\times l1},v_{2}^{y\times l2},...,v_{x}^{y\times lx}\}$, hence the concluding feature space V holds the dimensionality of y×(l1+l2+...+lx), while this kind of simple concatenation procedure frequently enhances the classification performance instead of using the single feature. In the feature embedding, a weak feature may affect the classification performance of other good features. In this case, the weighted feature embedding layer has been introduced to individually compute the cross-entropy loss of every feature, as well as to upgrade the allocated parameters via gradient descent optimizer and softmax function to reduce the cross-entropy loss, as shown in Figure 8. Recently, the gradient descent optimization technique (GDOT) achieved better performance in deep learning. In this research methodology, GDOT is applied for back-propagation to upgrade the weights and biases. The convoluted details of back-propagations can be obtained from [39]. In Figure 8, deep neural network models, namely VGG-19 and Inception-v3, with bottleneck features adopted with dimensions ${\overset{\wedge }{\underset{}{D}}}_{1},{\overset{\wedge }{\underset{}{D}}}_{2}$, respectively, are shown. Hence, the estimated feature vectors are joined together through a product $v=\prod_{j=1}^{2}\overset{\wedge }{D_{j}}$ and fed into the softmax classifier for the final decision.

## 3. Experimental Setup 

In the experiments, Intel (R) Xeon CPU e5-2683 v3@2.00Ghz (Chongqing University, Chongqing, China) was used with the memory of 64G. The GPU was NVIDIA TESLA K80 with the graphics memory of 12G. The platform for experiments was arranged with Opencv3.4, CUDAToolkit9.0, Anaconda3.4, and 64-bit ubuntu14.04. A deep learning framework with tensorflow1.9 was selected for the training and implementation of the proposed network.

## 4. Results and Discussion 

In the experiment, two publicly available datasets with a total number of 237 fundus images were applied for the analysis of the proposed approach. The E-Ophtha dataset subset “E-Ophtha-MA” holds 148 microaneurysms images, while DIARETDB1 contains 89 fundus photographs.

In the phase of training, fundus patches taken from 70 photographs specified for MA signs were collected from DIARETDB1 and used for training purposes; testing was performed on the remaining fundus images for validation. The total number of microaneurysms patches was 15,283 and non-microaneurysms patches were 45,360. The training process included zero to a hundred epochs, where error rate and accuracy were recorded with the use of a validation set and the time of completion process was noted as 248 min and 14 s. Table 1 provides detail about the division of MA and non-MA sign patches for training, testing, and validation. 

The evaluation performance was based on evaluation metrics [40,41], shown with formulas in Table 2.

**Figure 9:**
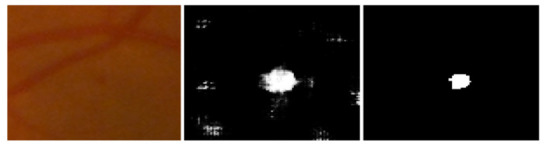
Left to right, stepwise microaneurysms detection: original image, MA sign before optimization, MA sign after optimization.

For the performance analysis of the proposed methodology, every patch-based image is analyzed and the extracted features were embedded by the feature embedding approach to merge the features obtained from different CNN models for the improvement of classification accuracy. Furthermore, to reduce the cross-entropy loss, using a back-propagation procedure was adopted based on a gradient descent optimizer to update the weights and bias. Figure 9 shows the MA sign detection with high accuracy through gradient descent optimization based on the feature embedding approach.

**Figure 10:**
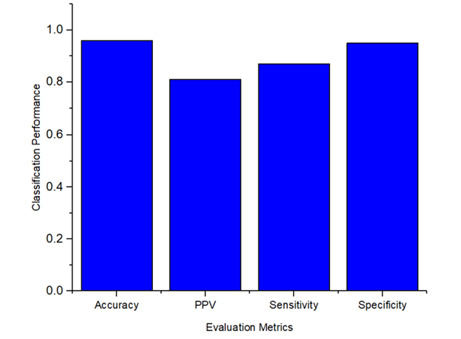
Microaneurysms sign classification performance.

The overall performance of the proposed methodology to detect and classify the microaneurysms was calculated for the evaluation metrics, including specificity as 0.95, sensitivity as 0.87, PPV as 0.81, and accuracy as 0.96. To provide a better understanding, Figure 10 demonstrates the classification performance results of MA signs.

**Figure 11:**
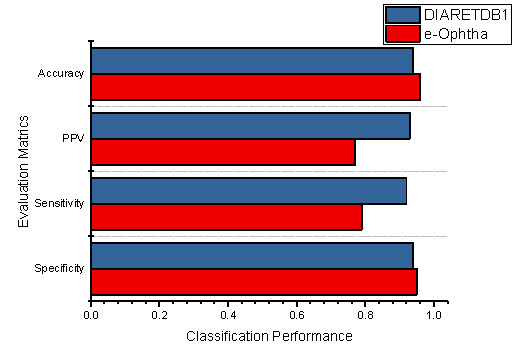
The performance evaluation of the proposed method using both datasets.

Additionally, the classification performances of the individual datasets are also demonstrated in Figure 11, which shows the specificity, sensitivity, PPV, and accuracy of the proposed methodology using DIARETDB1 and E-Ophtha datasets.

The total accuracy of the E-Ophtha dataset improved 0.02% compared to the DIARETDB1 dataset; similarly, 0.01% improvement was noticed in specificity. Furthermore, it was noticed that the proposed methodology obtained better classification results than the existing techniques. However, Table 3 demonstrates the comparative performance of our methodology against the literature study.

According to the comparative performance of the literature study, S. Joshi and P. Karule [30] achieved better sensitivity results but with a lower value of specificity. Furthermore, the existing methodologies presented a weaker performance in a few studies due to the absence of preprocessing techniques, the choice of traditional approaches, and utilization of less effective versions of CNN models for the feature extractions. However, the proposed methodology achieved better classification results than other existing studies. Furthermore, it was noticed that our model with a feature embedding approach obtained better classification performance. In the proposed framework, a feature embedding approach concatenated the features and was further optimized with the gradient descent optimization approach by updating weights and bias, and, finally, the softmax function classified the features with microaneurysms signs and non-microaneurysms. The performance evaluation results show that the proposed methodology outperformed the other existing approaches. 

## 5. Conclusions

In this paper, a deep neural network model was designed to analyze the microaneurysms for diabetic retinopathy through fundus photographs. The significance of this approach is in its ability to obtain features from two different DNNs, and then the most important technique is applied after feature extraction to concatenate the extracted features in order to improve the classification performance, which is known as feature embedding. Moreover, a gradient descent optimizer is implemented to reduce the cross entropy loss for the enhancement of accuracy. Finally, microaneurysms images are separated from non-microaneurysms images through a softmax classifier. Overall, the obtained results presented through the evaluation performance metrics show that the microaneurysms classification using feature embedding approach outperformed the other existing approaches.

## Figures and Tables

**Table 1:** Details of microaneurysms sign and non-microaneurysms sign patches.

	Microaneurysms Sign Patches	Non-Microaneurysms Sign Patches
Training Phase	10,433	28,367
Validation Phase	2496	9973
Testing Phase	2354	7020
Total number of patches	15,283	45,360

**Table 2:** Performance evaluation metrics.

Evaluation Metrics	Definition	Formula
Accuracy	The accuracy of a test is described as the ability to differentiate the patient and healthy cases correctly. To estimate the accuracy of a test, we should calculate the proportion of true positives and true negatives in all evaluated cases.	$\mathrm{Accuracy}=\frac{TN+TP}{TN+TP+FN+FP}$
Positive predictive values (PPV)	Probability of having the target condition given a positive test result	PPV=TP/(TP+FP)
Sensitivity	The sensitivity of a test shows the ability to determine the patient cases correctly. To estimate it, we should calculate the proportion of true positive in patient cases.	Sensitivity=TP/(TP+FN)
Specificity	The specificity of a test represents the ability to determine the healthy cases correctly. To estimate it, we should calculate the proportion of true negative in healthy cases.	Specificity=TN/(TN+FP)

**Table 3:** Overall microaneurysms classification performance of the proposed approach against literature study.

Methods	Datasets	Sensitivity	Specificity	Accuracy
Harangi et al. [40]	E-Ophtha-MA	0.64	0.88	0.69
Eftekhari et al. [41]	E-Ophtha-MA	0.77	-	-
T. Melo et al. [31]	E-Ophtha-MA	0.64	-	0.83
S. Joshi and P. Karule. [30]	E-Ophtha-MA	0.83	0.82	-
	DIARETDB1	0.89	0.91	0.92
Haiying Xia et al. [42]	E-Ophtha-MA	0.71	-	-
Proposed Methodology	E-Ophtha-MA	0.87	0.95	0.96
	DIARETDB1	0.92	0.94	0.94

## Data Availability

The data used in the experiment to support the findings of the proposed framework are available at the following links: E-Ophtha-MA. Available online: http://www.adcis.net/en/Download-Third-Party/E-Ophtha.html (8 August 2018). DIARETDB1. Available online: http://www.it.lut.fi/project/imageret/diaretdb1/index.html (8 August 2018).
